# Central pontine myelinolysis: a rare finding in hyperosmolar hyperglycemia

**DOI:** 10.3389/fneur.2023.1216328

**Published:** 2023-10-24

**Authors:** Hui-Ling Qu, Xiao-Yu Sun, Ying-Jie Dai

**Affiliations:** Department of Neurology, The General Hospital of Northern Theater Command, Shenyang, China

**Keywords:** central pontine myelinolysis, hyponatremia, hyperosmolar, osmotic demyelination syndrome, blood glucose

## Abstract

Central pontine myelinolysis (CPM) is a heterogeneous nervous system disease of pontine demyelination, usually caused by rapid correction of hyponatremia. In the present study, we report a unique case of a 46-year-old man with a hyperglycemic state complicated with CPM. MRI demonstrated a high signal on T2 and symmetric restricted diffusion in the pontine. In conclusion, the clinical case described confirmed that the hyperosmolar state inherent in hyperglycemia was a likely cause of CPM.

## 1. Introduction

Osmotic demyelination syndrome (ODS) is a rare complication of hyperglycemia and refers to central pontine myelinolysis and extrapontine myelinolysis manifesting with quadriparesis and neurocognitive changes with characteristic changes in magnetic resonance imaging (MRI) (1). The clinical manifestations range from low level of consciousness to dysarthria and then to quadriplegia (2). ODS is usually associated with myelin destruction in the central pontine basement, also known as central pontine myelinolysis (CPM). Here, we report a unique case of a 46-year-old man with a hyperglycemic state complicated with CPM.

## 2. Case report

**Figure 1:**
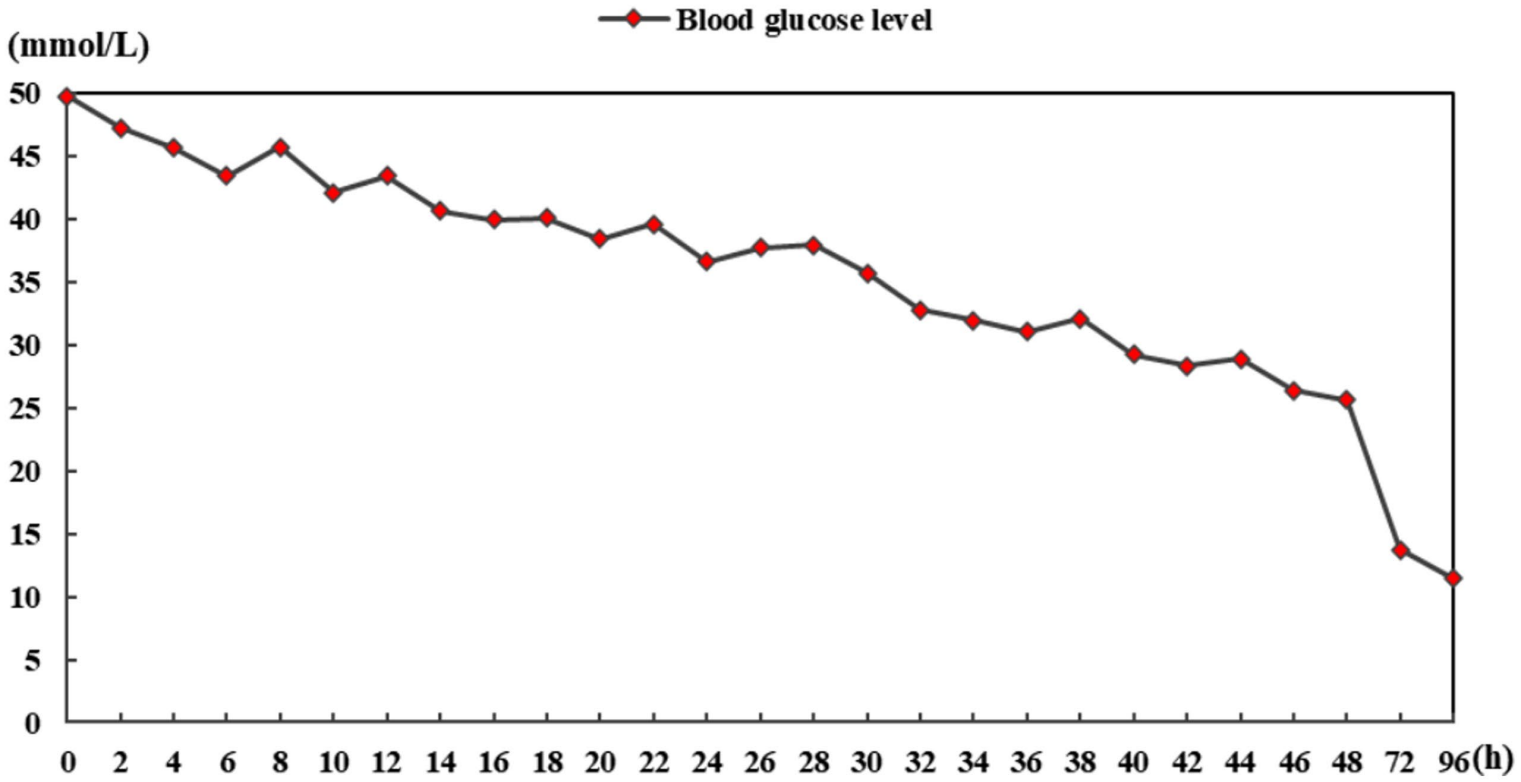
Blood glucose level chart.

**Figure 2:**
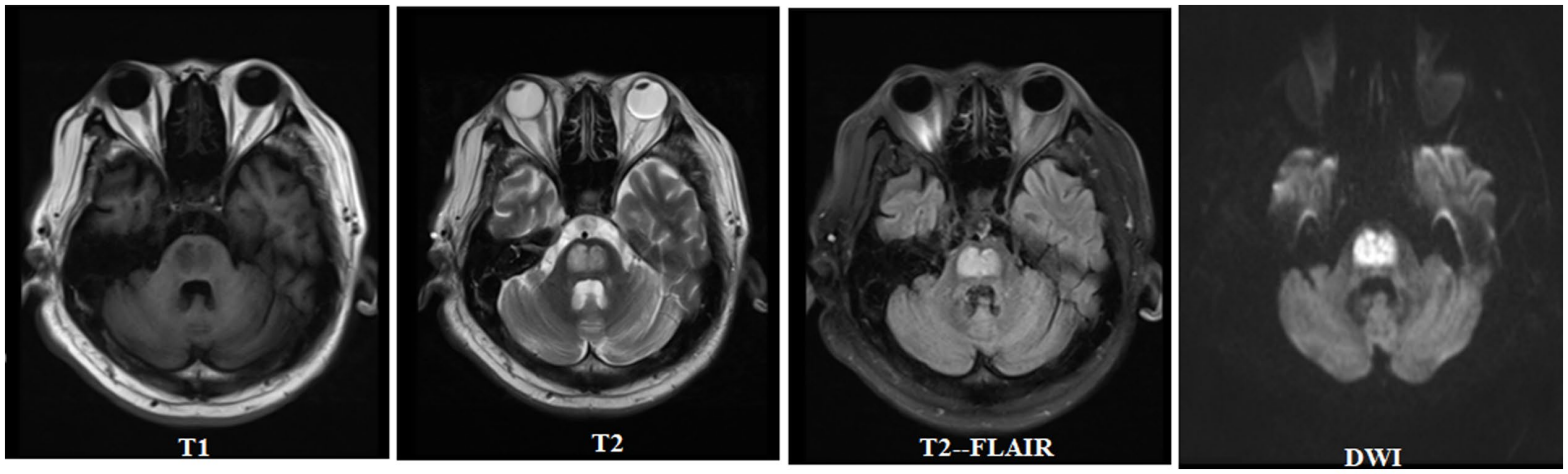
Central pontine myelinolysis in hyperglycemia.

A previously healthy 46-year-old man presented with limb weakness and dysarthria for 20 days. There was no history of malnutrition, a known diagnosis of diabetes, alcohol abuse, or smoking. Medical examination showed the patient presented with a body temperature of 36.6°C, respiratory rate of 18 breaths/min, normal resting heart rate of 76 beats/min, and blood pressure of 116/68 mm Hg. No abnormalities were found during heart and lung auscultation, and the patient presented with a soft abdomen and no swelling in either lower limb. A neurological examination revealed that he had mild dysarthria. His muscle tone was normal, with bilateral severe limb weakness and power was grade 2–3 in all of his limbs. Limb reflexes were mildly decreased and plantar flexor and sensation were normal. The blood results showed the following a blood glucose level of 49.7 mmol/L (894.6 mg/dL) and a normal range of 4–7 mmol/L. The hemoglobin A1c was 16.34%, 3-hydroxybutyric acid was 0.5 mmol/L, and he had 1+ ketone (20–29 mg/dL) in his urine. Notably, at presentation, his serum sodium was 128 mmoL/L (128 mEq/L), with a normal range of 133–146 mmol/L, but his serum potassium was markedly low at 2.8 mmol/L (2.8 mEq/L), with a normal range of 3.5–5.0 mmol/L. The calculated serum osmolality was 325 mosm/kg. Normal saline (0.9% NaCl) and insulin therapy (0.1 U/kg/h) were started and provided symptomatic treatment with potassium and sodium supplementation. After 6 h of admission, his blood glucose was 43.4 mmol/L (781.2 mg/dL) and serum sodium was 130 mmoL/L (130 mEq/L), whereas over the first 24 h of admission, his blood glucose reduced to 36.6 mmol/L (658.8 mg/dL), serum sodium increased to 135 mmoL/L (135 mEq/L), and 3-hydroxybutyric acid was 0.3 mmol/L. His blood glucose reduced to 25.6 mmol/L (460.8 mg/dL) over 48 h of admission and his serum sodium increased from 128 to 141 mmol/L (128 to 141 mEq/L). Over the first 48 h of admission, the patient’s serum potassium increased from 2.8 to 4.9 mmol/L (2.8 to 4.9 mEq/L), 3-hydroxybutyric acid was 0.05 mmol/L with negative ketonuria, and the calculated serum osmolality was 327 mOsm/kg. The blood glucose level is shown in Figure 1. In order to exclude peripheral nerve diseases, including acute inflammatory demyelinating polyneuropathy, lumbar puncture and electromyography were performed, and the results showed no abnormalities. The patient gradually became sleepy after admission, and the Glasgow Coma Score fluctuated between 13 and 14. However, there were no significant changes in his limb muscle power assessment. Because of the neurological symptoms, the patient subsequently underwent a cerebral MRI, which demonstrated features of CPM (Figure 2). The patient’s condition became stable and he was discharged to a rehabilitation institution. His blood glucose level was controlled with long-acting and short-acting insulin. In the following 6 months, his symptoms gradually improved and the power returned to grade 5 in all his limbs. The patient gradually resumed his daily activities without any residual symptoms (modified Rankin Scale [mRS] score = 0). Considering the clinical improvement of the patient and the absence of new neurological impairments, no repeated brain imaging was performed.

## 3. Discussion

CPM is a heterogeneous nervous system disease of pontine demyelination, usually caused by rapid correction of hyponatremia, and the correction speed exceeds 9 mmol/L/24 h (3, 4). In addition to rapidly correcting hyponatremia, other factors may be chronic alcoholism, hypernatremia, hypokalemia, hypophosphatemia, anorexia nervosa, and diabetes (5). Although rapid correction of hyponatremia is often considered a cause of CPM, considering the speed of initial serum sodium and electrolyte correction, our patient was unlikely to undergo rapid correction. Although the patient reported here presented with a serum sodium growth rate of 6.5 mmol/L/24 h, which did not exceed 9 mmol/L/24 h, we suggest that the inherent hypertonic state of hyperglycemia was more likely the cause of CPM. However, there are limited reports of associations between central pontine myelinolysis and derangement of glucose. It is reported that before the treatment of hyperglycemia and hyperosmolality (6, 7), there were cases of CPM, and our patient’s CPM may also have been caused by hyperosmolality before correction. Several reports have described patients with CPM in association with a hyperosmolar hyperglycemic state or its correction (8–17). Since there was no obvious change in osmotic pressure before and after correction, we consider that the CPM of our patient was related to the hyperosmolar state inherent in hyperglycemia. A diagnosis of CPM secondary to hyperglycemia was made.

Hyperglycemia and diabetes ketoacidosis are rare causes of CPM, and its physiopathology is uncertain (12). The pathophysiology of CPM may involve the sudden contraction of brain cells, especially oligodendrocytes, and demyelination caused by a rapid increase of serum osmotic pressure due to rapid correction of hyponatremia. Two theories have been proposed to explain oligodendrocyte contraction and myelinolysis, which are sometimes related to the rapid increase of serum osmotic pressure, whether due to the increase of sodium or other reasons, such as hyperglycemia: (i) local inflammatory demyelination caused by blood–brain barrier damage and (ii) oligodendrocyte apoptosis caused by hypertonic stress caused by changes in serum osmotic pressure, which occurs too fast to allow changes in specific molecules (11, 16). Research has shown that long-term episodes of severe hyperglycemia may lead to osmotic stress. Due to the poor control of diabetes, the unstable fluctuation of blood glucose may further lead to an unstable osmotic environment and the inability of normal cell compensation (18). MRI changes related to ODS typically include high signal lesions on T2-weighted MRI. The extension of T2-weighted signals can be explained by demyelination and edema (19).

Brain MRI remains the preferred diagnostic mode for CPM, and as described in the MRI results of this case, CPM may display a high signal on T2 and symmetric restricted diffusion in the pontine (10, 20). Despite reports of asymmetric lesions, pontine lesions are usually symmetrical (21). Our patient displayed the typical radiological and clinical findings of pontine myelinolysis with hyperglycemia, which is a rare phenomenon. Effective specific treatment methods have not yet been determined for the management of ODS cases. Therefore, the current treatment model includes general supportive care and treatment for potential causes, which is everything we do to treat patients. Similarly, there is no clear recommendation for the optimal timing of MRI imaging. The degree of clinical features and/or radiological changes cannot reliably determine the prognosis. The results vary from recovery to near-normal functional levels and death. More than half of ODS cases have recovered well, and the mortality rate of ODS cases has decreased over time. Even with severe neurological manifestations, patients with ODS may have a good prognosis (22).

In conclusion, the clinical case described confirmed that the hyperosmolar state inherent in hyperglycemia was a likely cause of CPM. Clinicians should consider hyperglycemia in patients with diabetes mellitus who develop CPM.

## Data availability statement

The original contributions presented in the study are included in the article/supplementary material, further inquiries can be directed to the corresponding author.

## Ethics statement

Written informed consent was obtained from the individual(s) for the publication of any potentially identifiable images or data included in this article.

## Author contributions

Y-JD conceived the study. H-LQ and X-YS collected the data and drafted the manuscript. All authors contributed to the article and approved the submitted version.

## References

[1] King JD Rosner MH. Osmotic demyelination syndrome. Am J Med Sci. (2010) 339:561–7. doi: 10.1097/MAJ.0b013e3181d3cd78 20453633

[2] Laureno R Karp BI. Myelinolysis after correction of hyponatremia. Ann Intern Med. (1997) 126:57–62. doi: 10.7326/0003-4819-126-1-199701010-00008, PMID: 8992924

[3] Fitts W Vogel AC Mateen FJ. The changing face of osmotic demyelination syndrome: a retrospective, observational cohort study. Neurol Clin Pract. (2021) 11:304–10. doi: 10.1212/CPJ.0000000000000932, PMID: 34484930 PMC8382430

[4] Rodríguez-Velver KV Soto-Garcia AJ Zapata-Rivera MA Montes-Villarreal J Villarreal-Pérez JZ Rodríguez-Gutiérrez R. Osmotic demyelination syndrome as the initial manifestation of a hyperosmolar hyperglycemic state. Case Rep Neurol Med. (2014) 2014:652523. doi: 10.1155/2014/652523, PMID: 25431711 PMC4241748

[5] Chaudhary A Chaudhary A Yadav RS Shrestha Y Shah R. Pediatric osmotic demyelination syndrome in a case of type 1 diabetes mellitus with diabetic ketoacidosis. Clin Case Rep. (2022) 10:e05584. doi: 10.1002/ccr3.5584, PMID: 35340640 PMC8934147

[6] Bline K Singh D Poeppelman R Lo W O’Brien N. Extrapontine Myelinolysis and microhemorrhages: rare finding in pediatric diabetic ketoacidosis. Pediatr Neurol. (2018) 89:68–70. doi: 10.1016/j.pediatrneurol.2018.09.006, PMID: 30396831

[7] Saini M Mamauag MJ Singh R. Central pontine myelinolysis: a rare presentation secondary to hyperglycaemia. Singap Med J. (2015) 56:e71–3. doi: 10.11622/smedj.2015065, PMID: 25917480 PMC4415110

[8] Bonkowsky JL Filloux FM. Extrapontine myelinolysis in a pediatric case of diabetic ketoacidosis and cerebral edema. J Child Neurol. (2003) 18:144–7. doi: 10.1177/08830738030180021201, PMID: 12693785

[9] Burns JD Kosa SC Wijdicks EFM. Central pontine myelinolysis in a patient with hyperosmolar hyperglycemia and consistently normal serum sodium. Neurocrit Care. (2009) 11:251–4. doi: 10.1007/s12028-009-9241-9, PMID: 19565358

[10] Gonzalez Caldito N Karim N Gebreyohanns M. Teaching NeuroImage: central pontine Myelinolysis in diabetic ketoacidosis. Neurology. (2021) 97:e1971–2. doi: 10.1212/WNL.0000000000012301, PMID: 34078715

[11] Guerrero WR Dababneh H Nadeau SE. Hemiparesis, encephalopathy, and extrapontine osmotic myelinolysis in the setting of hyperosmolar hyperglycemia. J Clin Neurosci. (2013) 20:894–6. doi: 10.1016/j.jocn.2012.05.045, PMID: 23477877

[12] Matías-Guiu JA Molino AM Jorquera M Jiménez R Ruiz-Yagüe M. Pontine and extrapontine myelinolysis secondary to glycemic fluctuation. Neurologia. (2016) 31:345–7. doi: 10.1016/j.nrl.2014.06.005, PMID: 25440064

[13] McComb RD Pfeiffer RF Casey JH Wolcott G Till DJ. Lateral pontine and extrapontine myelinolysis associated with hypernatremia and hyperglycemia. Clin Neuropathol. (1989) 8:284–8. PMID: 2695277

[14] McKee AC Winkelman MD Banker BQ. Central pontine myelinolysis in severely burned patients: relationship to serum hyperosmolality. Neurology. (1988) 38:1211–7. doi: 10.1212/WNL.38.8.1211, PMID: 3399069

[15] O’Malley G Moran C Draman MS King T Smith D Thompson CJ. Central pontine myelinolysis complicating treatment of the hyperglycaemic hyperosmolar state. Ann Clin Biochem. (2008) 45:440–3. doi: 10.1258/acb.2008.007171, PMID: 18583636

[16] Sharma C Kumawat BL Panchal M Shah M. Osmotic demyelination syndrome in type 1 diabetes in the absence of dyselectrolytaemia: an overlooked complication? BMJ Case Rep. (2017) 2017:bcr2016219148. doi: 10.1136/bcr-2016-219148 PMC5747637 28500261

[17] Sivaswamy L Karia S. Extrapontine myelinolysis in a 4 year old with diabetic ketoacidosis. Eur J Paediatr Neurol. (2007) 11:389–93. doi: 10.1016/j.ejpn.2007.02.017, PMID: 17425961

[18] Donnelly H Connor S Quirk J. Central pontine myelinolysis secondary to hyperglycaemia. Pract Neurol. (2016) 16:493–5. doi: 10.1136/practneurol-2016-001389, PMID: 27407176

[19] Murase T Sugimura Y Takefuji S Oiso Y Murata Y. Mechanisms and therapy of osmotic demyelination. Am J Med. (2006) 119:S69–73. doi: 10.1016/j.amjmed.2006.05.010, PMID: 16843088

[20] Ruzek KA Campeau NG Miller GM. Early diagnosis of central pontine myelinolysis with diffusion-weighted imaging. AJNR Am J Neuroradiol. (2004) 25:210–3. PMID: 14970019 PMC7974598

[21] Bansal LR. Therapeutic effect of steroids in osmotic demyelination of infancy. Child Neurol Open. (2018) 5:2329048X1877057. doi: 10.1177/2329048X18770576, PMID: 29687030 PMC5903026

[22] Singh TD Fugate JE Rabinstein AA. Central pontine and extrapontine myelinolysis: a systematic review. Eur J Neurol. (2014) 21:1443–50. doi: 10.1111/ene.12571, PMID: 25220878

